# Treatment failure of *Helicobacter pylori* in primary care: a retrospective cohort study

**DOI:** 10.3399/BJGPO.2023.0252

**Published:** 2024-07-10

**Authors:** Gertrude van den Brink, Lieke M Koggel, Joris JH Hendriks, Mark GJ de Boer, Peter D Siersema, Mattijs E Numans

**Affiliations:** 1 Department of Public Health and Primary Care, Leiden University Medical Centre, Leiden, the Netherlands; 2 Department of Gastroenterology and Hepatology, Radboud Institute for Health Sciences, Radboud University Medical Centre, Nijmegen, the Netherlands; 3 Department of Infectious Diseases & Department of Clinical Epidemiology Leiden University Medical Centre, Leiden University Centre for Infectious Diseases, Leiden, the Netherlands; 4 Department of Gastroenterology and Hepatology, Erasmus University Medical Centre, Rotterdam, the Netherlands

**Keywords:** Helicobacter pylori, treatment failure, primary health care

## Abstract

**Background:**

Owing to increasing antibiotic resistance, the worldwide efficacy of *Helicobacter pylori* (HP) eradication treatment has decreased.

**Aim:**

To determine antimicrobial resistance of HP in primary care.

**Design & setting:**

Retrospective cohort study using real-world routine healthcare data from 80 general practices in the Netherlands.

**Method:**

Patients with International Classification of Primary Care (ICPC) codes for gastric symptoms or Anatomical Therapeutic Chemical (ATC) codes for acid inhibition in the period 2010–2020 were selected. Main outcomes were antimicrobial resistance of HP, defined as the prescription of a second eradication treatment within 12 months, and clinical remission of gastric symptoms, defined as no usage of acid inhibition 1 year following eradication therapy.

**Results:**

We identified 138 455 patients with gastric symptoms and/or acid inhibition use (mean age 57 years [standard deviation 18.2 years], 43% male). A total of 5224 (4%) patients received an HP eradication treatment. A second treatment was prescribed to 416 (8%) of those patients. From these, 380 patients received amoxicillin–clarithromycin, 16 amoxicillin–metronidazole, and 11 clarithromycin–metronidazole as first regimen and were considered antimicrobial resistant. We observed a 0.8% increment per year of patients requiring a second eradication treatment (*P* = 0.003, 95% confidence interval = 0.33 to 1.22). After successful eradication, 2329/4808 (48%) patients used acid inhibition compared with 355/416 (85%) patients following treatment failure (*P*<0.001).

**Conclusion:**

Antimicrobial treatment is not successful in almost one-tenth of HP infections in primary care after a first treatment containing clarithromycin and/or metronidazole. Although the treatment failure rate is not as high as reported in secondary care, the increasing trend is concerning and may require revision of the current guidelines.

## How this fits in

It is recommended to treat all *Helicobacter pylori (*HP) infections regardless of symptoms to prevent serious complications such as peptic ulcer disease and gastric cancer. Increasing antimicrobial resistance rates of HP have been reported in cultures in secondary care, which may influence treatment success. Our study indicates that the HP antimicrobial resistance rate in primary care is not as high and not rising as fast as reported in secondary care. However, a slowly increasing trend over the years still raises concern. Therefore, revision of primary care guidelines on antimicrobial regimens for HP should be considered, also taking feasibility and compliance into account.

## Introduction

*Helicobacter pylori* is a gram-negative bacterium that colonises the stomach^1^ and remains in the mucosa during life, unless it is eradicated.^2^ HP is usually acquired during childhood.^3^ About half of the world’s population is infected with HP. However, the reported infection rates range from 10%–90%, and are higher in low-income countries compared with high-income countries.^4^ In the Netherlands, the reported prevalence of HP is 35%.^4,5^ Although HP always causes gastritis, 50%–70% of patients do not have gastric symptoms.^6–9^ For several years, HP has been considered an infectious disease, which implies treatment of all infected patients, to prevent serious complications.^7,10^ HP infection can cause severe complications such as peptic ulcer disease (10%–20%), mucosa-associated lymphoid tissue (MALT) lymphoma, and (non-cardia) gastric adenocarcinoma (1%–2%).^11^ HP is estimated to be responsible for 85% of (non-cardia) gastric cancer and eradication reduces the risk of gastric cancer.^11–15^

The worldwide increasing antimicrobial resistance of HP is concerning and raises the question whether treatment guidelines should be updated.^16–18^ Antimicrobial resistance in HP is, however, unlike some other clinically relevant bacterial species, not systematically monitored.^19,20^ Dutch studies regarding antimicrobial resistance are scarce and concern susceptibility testing in biopsy specimens in secondary care centres, and are therefore not representative for primary care.^19–21^ For example, a recent study showed an increase in resistance to clarithromycin (7%–40%) and metronidazole (14%–45%) from 2010–2019.^21^ To the best of our knowledge, no direct resistance data about HP resistance in the primary care setting are available (inter)nationally. Therefore, the primary aim of this study was to determine antimicrobial resistance development, by measuring eradication success of HP in primary care in the Netherlands as a proxy measure. As a secondary outcome, we studied clinical remission of gastric symptoms.

## Method

Susceptibility tests are not performed in primary care. Therefore, we formulated a proxy for antimicrobial resistance based on the available routine healthcare data. Data on HP eradication treatments, HP tests, and the presence of gastric symptoms were collected using drug prescriptions, laboratory results, GP consult registrations, and medical histories.

Real-world pseudonymised data from general practices in the province of South Holland, the Netherlands, was made available by the Extramural Leiden University Medical Centre Academic Network (ELAN). The ELAN database contains 529 933 adult patients (aged ≥18 years) from 80 general practice centres with 2–10 practising GPs. All patients were included unless they opted out (±0.2%). All the different Dutch urban areas are represented within ELAN.

### Data collection

Data on patient characteristics, medical history, HP tests, and drug prescriptions between 2010 and 2020 were available. Medical conditions were displayed as International Classification of Primary Care (ICPC) codes and drug prescriptions as Anatomical Therapeutic Chemical (ATC) codes. Patients aged between 18 and 70 years who had presented with gastric symptoms or used acid inhibition in the period 2010–2020 were selected based on ICPC and ATC codes (see Supplementary Table S1). If available, we analysed the HP tests performed in laboratories connected to the GP's electronic medical records. In case of multiple tests with a known outcome on the same day, stool antigen tests were preferred over serology, and serology over faeces polymerase chain reaction (PCR).

### HP eradication therapy

If a patient received eradication treatment in 2011–2019, they were classified as having a ‘HP infection’. Data from 2010 were used to determine drug use in the year before eradication therapy and data from 2020 to determine succeeding treatments. HP eradication treatment was defined as the prescription of either a proton pump inhibitor (PPI) with at least two types of antibiotics initiated simultaneously or a fixed-dose combination (triple therapy). ATC codes used can be found in Supplementary Table S1.

HP eradication therapy was considered successful if no second eradication treatment was prescribed >42 days and <12 months after initiation of HP treatment. Treatment failure, defined as the prescription of a second eradication treatment within 12 months, was used as a proxy of antimicrobial resistance. The types of antibiotics prescribed as first eradication treatment were evaluated for resistance rates.

The end dates of antibiotic prescriptions were calculated using the start date, dosage, and usage frequency. If the prescribed frequency was not (exactly) provided, it was replaced by the standard instruction for use of this drug as described in Supplementary Table S1. Duration of antibiotic use was extracted, and classified as 7, 10, 14, or 21 days±1.5 days.^22^ Durations that did not fulfil these criteria were considered missing.

### Acid inhibition

The use of acid inhibition after successful eradication treatment was evaluated to determine clinical remission of gastric symptoms (secondary outcome). A new or refill prescription of a PPI or H_2_-receptor antagonist (H_2_RA) between 3 and 12 months after successful eradication treatment was interpreted as persistent gastric symptoms. Patients with no new prescription were considered to be in clinical remission.

### Antibiotic use

We analysed antibiotic use in the year before the first eradication treatment as this may influence success of eradication. Antibiotics were subdivided into groups based on their structure and mechanism of antimicrobial activity.

### Risk factors for treatment failure

Risk factors for HP treatment failure were analysed by successful versus failed first eradication treatment. The following variables were included: patient characteristics (sex, age, body mass index [BMI], smoking, and alcohol use), comorbidities (ICPC codes for diabetes mellitus, heart failure, ischaemic heart disease, hypertension, cerebrovascular accident, and chronic obstructive pulmonary disease [COPD]), duration of eradication treatment, acid inhibition, and antibiotic use (β-lactams, tetracyclines, macrolides, quinolones, and other antibiotics defined as metronidazole and rifamycin) in the year before the first eradication treatment.

### Statistical analysis

Data were presented as mean ± standard deviation (SD; normally distributed) and median and interquartile range (non-normally distributed). Treatment success was reported as percentages and absolute values per year. χ^2^ testing was performed to compare categorical variables. An autoregressive integrated moving average model was used to analyse the course of treatment success over the years. A generalised logistic regression model was used to determine risk factors of treatment success. Variables with a *P*-value <0.2 in the univariable analysis were included in the multivariable analysis. A backwards model was used to stepwise eliminate the variables with the highest *P*-value until all variables in the model had a *P*-value <0.05. All analyses were performed using IBM SPSS Statistics (version 27).

## Results

**Figure 1.**
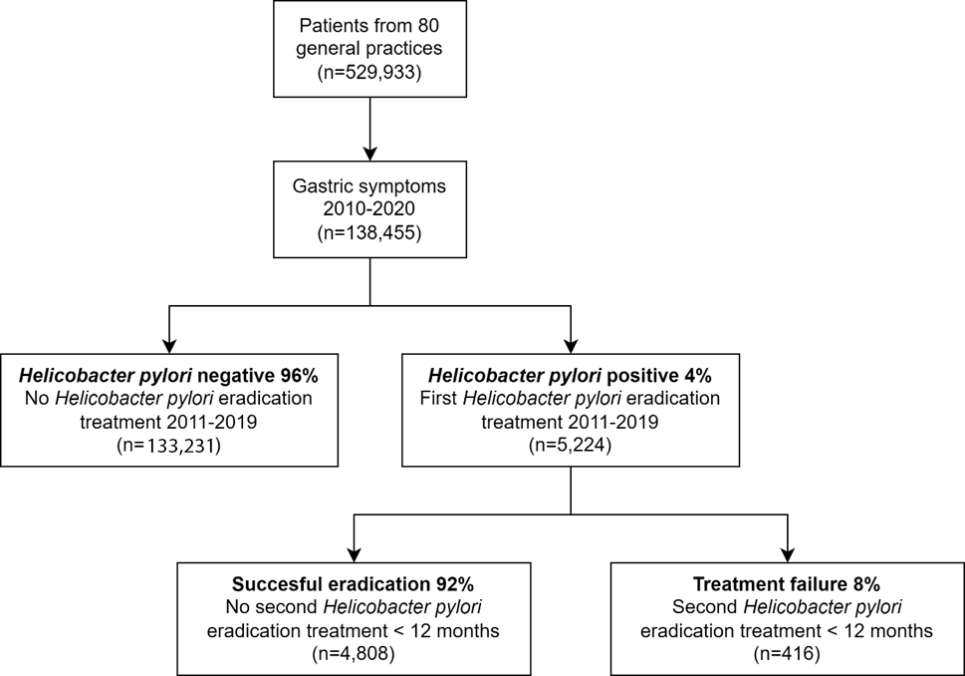
Patient selection

We identified 138 455 adult patients with gastric symptoms or acid inhibition in the years 2010–2020 from 80 general practices (Figure 1). Mean age was 57 years (SD 18 years) of whom 43% were male. Alcohol use was registered in 23 991 (17%) of patients and 18 204 (13%) were registered as smoker. Mean BMI was 29 kg/m^2^ (SD 6 kg/m^2^). All baseline characteristics are shown in Table 1.

**Table 1.** Baseline characteristics

Characteristic	Gastric symptoms (*n* = 138 455)	HP positive (*n* = 5224)	Successful treatment (*n* = 4808)	Treatment failure (*n* = 416)
Male, *n* (%)	60 072 (43)	2094 (40)	1940 (40)	154 (37)
Age, years, mean (SD)	56.7 (18.2)	53.6 (15.6)	53.8 (15.7)	50.4 (14.7)
BMI, kg/m2, mean (SD)^a^	28.5 (5.7)	28.7 (5.8)	28.6 (5.7)	29.5 (6.4)
Smoking, *n* (%)^b^				
Yes	18 204 (13)	725 (14)	658 (14)	67 (16)
No	55 (0)	2 (0)	2 (0)	0 (0)
Alcohol, *n* (%)^c^				
Yes	23 991 (17)	610 (12)	585 (12)	25 (6)
No	4522 (3)	217 (4)	198 (4)	19 (5)
Comorbidity, *n* (%)				
Diabetes mellitus	9491 (7)	397 (8)	367 (8)	30 (7)
Heart failure	5269 (4)	147 (3)	136 (3)	11 (3)
Ischaemic heart disease	5546 (4)	180 (3)	169 (4)	11 (3)
Hypertension	24 298 (18)	908 (17)	836 (17)	72 (17)
Cerebrovascular accident	2695 (2)	81 (2)	77 (2)	4 (1)
COPD	3866 (3)	102 (2)	97 (2)	5 (1)
**Drug use in year before HP eradication,** ***n*** **(%)**				
Acid inhibition^d^	NA	2961 (57)	2738 (57)	223 (54)
Proton pump inhibitor	NA	2707 (52)	2505 (52)	202 (49)
H2-receptor antagonist	NA	472 (9)	426 (9)	46 (11)
Antibiotics^d^	NA	1232 (24)	1106 (23)	126 (30)
β-lactams	NA	708 (14)	631 (13)	77 (19)
Tetracyclines	NA	329 (6)	298 (6)	31 (7)
Macrolides	NA	264 (5)	217 (5)	47 (11)
Quinolones	NA	163 (3)	151 (3)	12 (3)
Other^e^	NA	102 (2)	90 (2)	12 (3)

^a^Missing, *n* = 82 432 (60%). ^b^Missing, *n* = 120 196 (87%). ^c^Missing, *n* = 109 941 (79%). ^d^For the subcategories, combinations (for example, double users) are included in the numbers. ^e^Other included metronidazole and rifamycin. BMI = body mass index. COPD = chronic obstructive pulmonary disease. HP *= Helicobacter pylori*. NA = not applicable. SD = standard deviation.

One or more of the available HP tests were registered in 7666 patients (see Supplementary Table S2). Test results were available for 74%. The tests mostly concerned stool antigen tests (*n* = 4044, 53%) or serology (*n* = 3561, 46%), followed by faecal PCR (*n* = 61, 1%). More than half of the HP tests showed a negative result (*n* = 4176, 54%), and almost one in five a positive result (*n* = 1489, 19%). The remaining test results were unknown (*n* = 2001, 26%).

### HP eradication therapy

**Figure 2.**
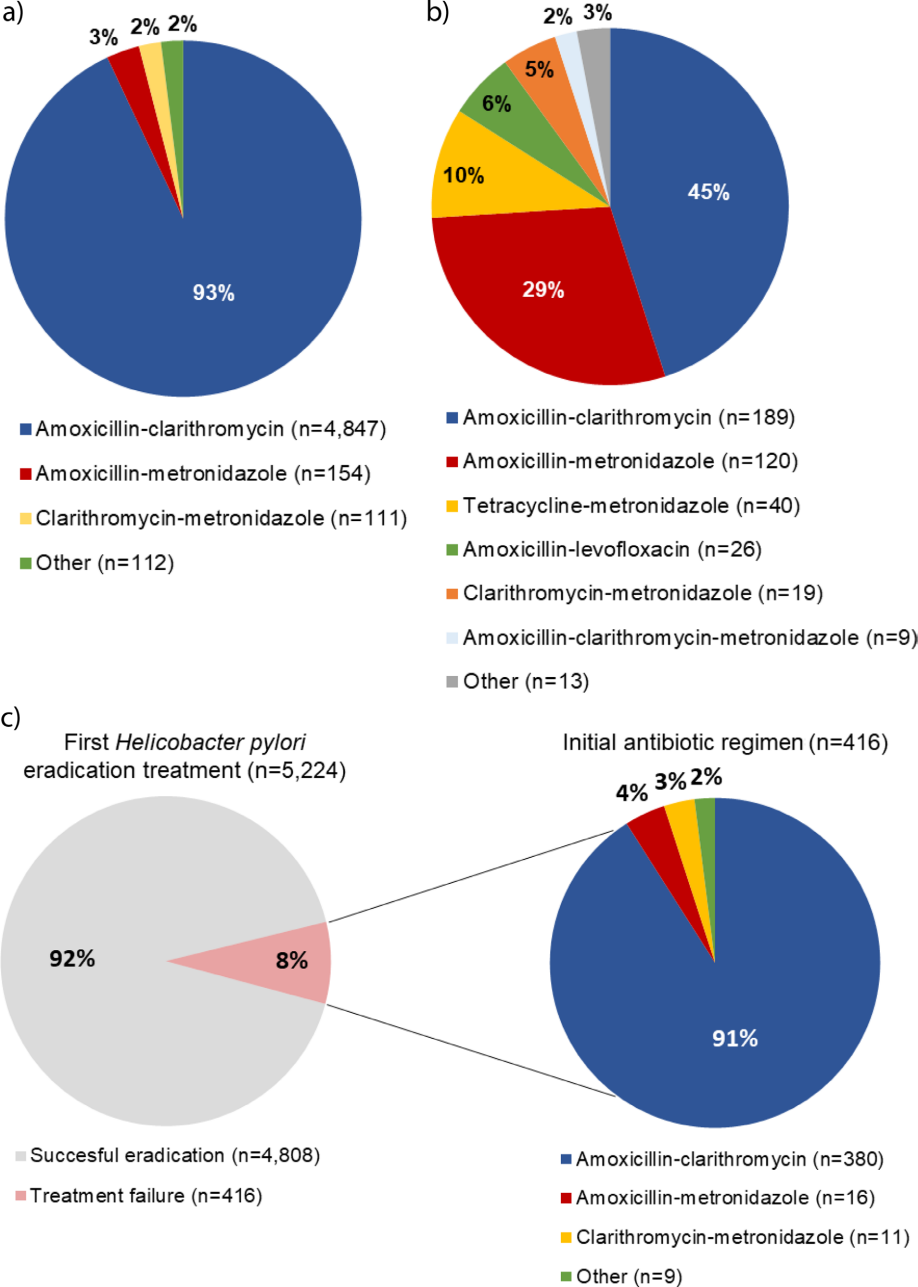
*Helicobacter pylori* (HP) eradication treatments: a) first HP eradication treatment; b) second HP eradication treatment; and c) initial antibiotic regimes of patients that received a second eradication treatment

A total of 5224 (4%) patients received a first eradication treatment between 2011 and 2019 and were therefore considered HP positive. Registration of a HP test before start of the eradication treatment was accessible in 1921/5224 (37%) of patients. The antimicrobial treatment combinations amoxicillin–clarithromycin, amoxicillin–metronidazole, and clarithromycin–metronidazole were prescribed in 4847 (93%), 154 (3%), and 111 (2%) of first eradication treatments, respectively (Figure 2 and Supplementary Table S3). Most treatments were prescribed for a period of ≤7 days (*n* = 4362, 83%) (see Supplementary Table S4).

### Treatment failure

**Figure 3.**
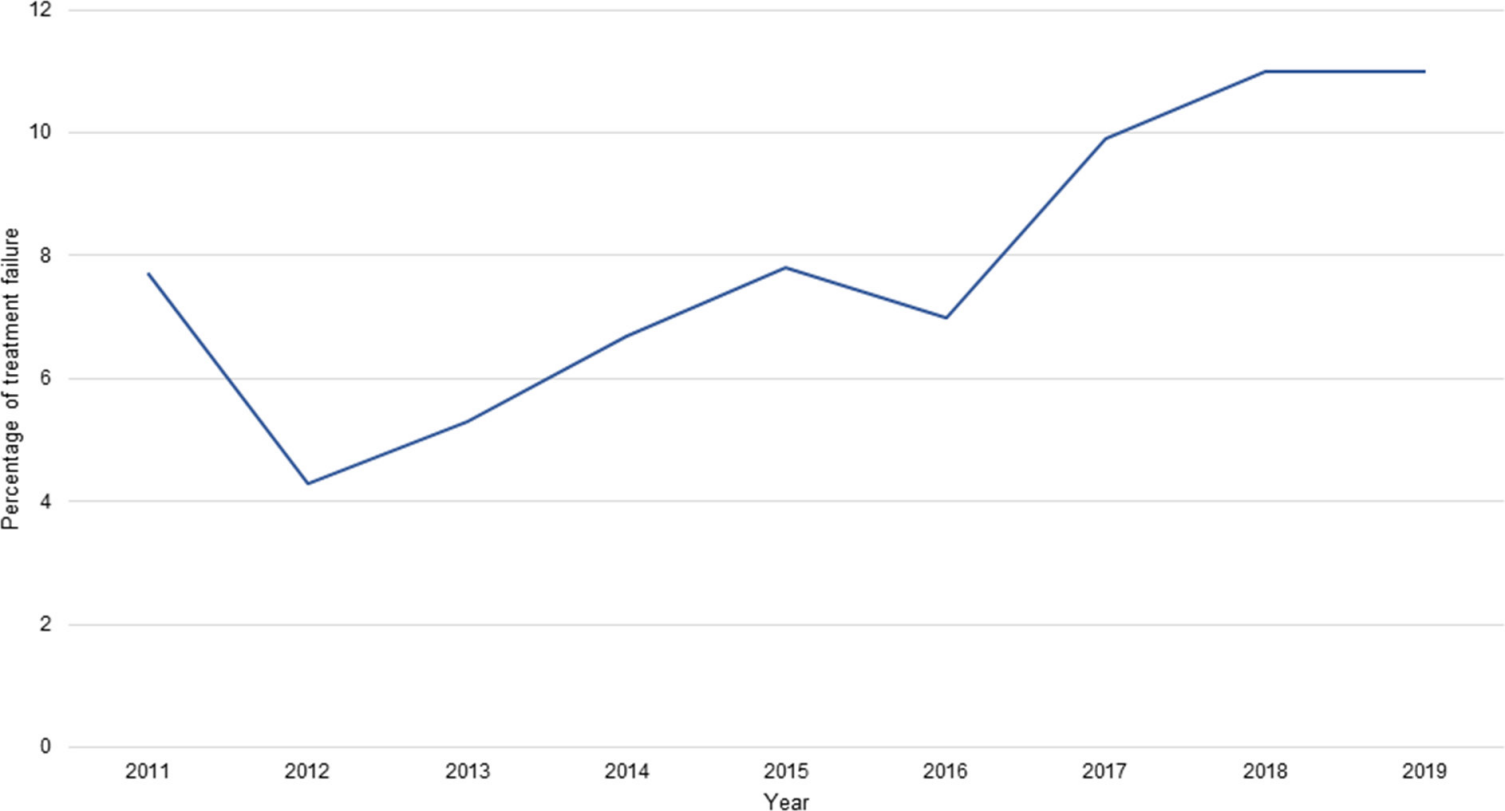
Percentage of *Helicobacter pylori* treatment failure over the years 2011–2019

A total of 416/5224 (8%) patients received a second eradication treatment within 1 year after the first eradication treatment and were therefore considered antimicrobial resistant. A 0.8% increment per year of patients in need of a second eradication treatment was seen (*P* = 0.003, 95% confidence interval [CI] = 0.33 to 1.22). Figure 3 and Supplementary Table S5 show the course of treatment failure over the years 2011–2019.

The second eradication treatment mostly included the combination of amoxicillin–clarithromycin (*n* = 189, 45%), amoxicillin–metronidazole (*n* = 120, 29%) and tetracycline–metronidazole (*n* = 40, 10%) (Figure 2 and Supplementary Table S3). In 299 (72%) patients the treatment was prescribed for a period of ≤7 days (see Supplementary Table S4).

Of the patients who received amoxicillin–clarithromycin, amoxicillin–metronidazole, and clarithromycin–metronidazole as the first eradication treatment, a total of 380/4847 (8%), 16/154 (10%), and 11/111 (10%) patients, respectively, were prescribed a second eradication treatment and therefore considered antimicrobial resistant (Figure 2).

### Clinical remission

In the year before the first HP eradication treatment, 2961/5224 (57%) patients used acid inhibition, in 2489 (48%) patients a PPI, in 254 (5%) patients an H_2_RA, and in 218 (4%) patients both PPI and H_2_RA.

Between 3 and 12 months after successful eradication therapy, 2329/4808 (48%) patients were prescribed a new or refill prescription of acid inhibition and therefore were considered to have persistent gastric symptoms. In the treatment failure group this concerned 355/416 (85%) patients (*P*<0.001) (data not shown).

### Antibiotic use before eradication

A total of 1232/5224 (24%) patients used antibiotics in the year before the first HP eradication treatment. This included 1106/4808 (23%) patients in the successful eradicated group and 126/416 (30%) in the treatment failure group. β-lactams were most frequently used (*n* = 708, 14%), followed by tetracyclines (*n* = 329, 6%), and macrolides (*n* = 264, 5%). Detailed data about the previously prescribed antibiotic regimens can be found in Supplementary Table S6.

### Risk factors for treatment failure

Variables with substantial missing data (BMI), not fully registered data (smoking and alcohol use), or small numbers (heart failure, ischaemic heart disease, cerebrovascular accident, COPD, quinolones, and other antibiotic use in year before HP eradication therapy) were excluded from the analysis. Risk factors for treatment failure were age (odds ratio [OR] 0.99 increment per year, 95% CI = 0.98 to 0.99), duration of eradication therapy >7 days (OR 0.63, 95% CI = 0.44 to 0.90), and use of β-lactam or macrolides antibiotics in the year before eradication treatment (OR 1.36, 95% CI = 1.03 to 1.80 and OR 2.76, 95% CI = 1.95 to 3.91, respectively) (Table 2).

**Table 2.** Risk factors for *Helicobacter pylori* eradication treatment failure

	Univariable logistic regression			Multivariable logistic regression		
**Characteristic**	**OR**	**95% CI**	***P*-value**	**OR**	**95% CI**	***P*-value**
Female	1.151	0.935 to 1.416	0.184			
Age, increment per year	0.986	0.980 to 0.992	<0.001	0.986	0.979 to 0.992	<0.001
Diabetes mellitus	0.940	0.639 to 1.384	0.756			
Hypertension	0.994	0.763 to 1.296	0.967			
Duration eradication therapy >7 days	0.613	0.427 to 0.880	0.008	0.626	0.435 to 0.901	0.012
Acid inhibition used in year before eradication therapy	0.874	0.715 to 1.068	0.187			
β-lactams used in year before eradication therapy	1.504	1.158 to 1.952	0.002	1.359	1.029 to 1.796	0.031
Tetracyclines used in year before eradication therapy	1.219	0.830 to 1.790	0.313			
Macrolides used in year before eradication therapy	2.695	1.932 to 3.758	<0.001	2.758	1.946 to 3.908	<0.001

OR = odds ratio.

## Discussion

### Summary

This large cohort of primary care patients shows an estimated HP antimicrobial treatment failure rate, as proxy for antimicrobial resistance rate, of almost 10%. An increasing trend of HP treatment failure was observed over 10 years, with an overall yearly increment of 1%, which raises concern for coming decades. After successful HP eradication, significantly more patients were in clinical remission of gastric symptoms. Risk factors for antimicrobial resistance were lower age, shorter duration of eradication therapy, and use of β-lactam or macrolides antibiotics in the year before eradication treatment.

### Strengths and limitations

To our knowledge, this is the first study to evaluate HP eradication success in primary care. Previous studies focused on secondary care data only, which is expected to be less representative for the general population since persisting complaints or refractory symptoms after intervention are the main reason for referral. Moreover, other strengths of this study are the large population size and the use of real-world routine care data that facilitates a reliable representation of current clinical practice.

Our study is limited by its retrospective design and missing data on diagnostic tests for HP owing to the organisation of primary care medical records and privacy guidelines. This forced us to define HP-positive cases by the start of eradication treatment. Also, we may have missed patients who were HP positive who were promptly treated after referral to secondary care and patients who did test positive but refused treatment. In addition, treatment failure (need of second eradication treatment) was chosen as a proxy for antimicrobial resistance. We recognise that treatment failure is not only affected by resistance but also by multiple other factors, such as misdiagnoses, treatment compliance, and treatment regimen. The extent of misdiagnoses in our study is unknown owing to the missing data in HP tests. However, it is thought to be minimal as the primary care guideline only advises treatment after a positive test. Unfortunately, data concerning therapy adherence are not available when using real-world data. Although, treatment costs and adverse drug reactions could have hampered therapy adherence. We did describe and considered all used treatment regimens. Hence, for the purpose of this study, it was the best proxy of antimicrobial resistance. Furthermore, our analysis showed that prior use of antibiotics was associated with treatment failure, which strongly supports that antimicrobial resistance is expected to play a key role in treatment failure rates. As only data on prescribed drugs were available, we could not take over-the-counter antireflux drug use into consideration to determine remission of gastric symptoms. However, this concerned both the successful eradicated group and the treatment failure group, and is therefore unlikely to affect the difference between the groups. And last, registrations on patient characteristics, such as BMI, smoking behaviour, and alcohol consumption, which were only partly available, may have influenced our results.

### Comparison with existing literature

The 8% estimated resistance rate found in our study is quite reassuring, considering the (much) higher percentages (up to 45%) and faster increasing antimicrobial resistance percentages found in Dutch^21^ and international secondary care data.^18,23,24^ This difference can be explained by selection, that is, secondary care data comprise the patient group with persisting or refractory symptoms with presumably higher antimicrobial resistance rates. Unfortunately, no data concerning direct resistance rates in primary care were available for comparison. Which level of resistance is concerning depends on several factors, such as consequences of treatment failure, the time frame until discovery of treatment failure, and disadvantages of alternative treatments, and is often set around 10%.^25^ Our data suggest that antimicrobial resistance exists for clarithromycin and metronidazole containing regimens, which corresponds with (inter)national studies.^18,21,26^ Although the failed treatment regimens also contained amoxicillin, it is not expected that amoxicillin resistance is of major importance as a prior study on susceptibility testing in biopsy specimens showed relatively low resistance rates (0%–5%).^21^

The relatively slow increase in estimated resistance over the past decade (nearly 1% per year) is concerning for several reasons. First, this increase most likely reflects a true increase in resistance, because all other factors influencing treatment outcome (treatment adherence and treatment guidelines) did not change in the past decade. Second, if test and treatment strategies remain as they are now, it is likely that this trend will continue, with more treatment failure being observed in the future. Third, as HP infections are frequently treated in primary care, this concerns a relatively high number of medical consultations, which will grow with increasing resistance.^27^

Concerning treatment regimens, first eradication regimens were consistent with local guidelines, but in 45% of second eradication treatments, guidelines were not followed and again amoxicillin–clarithromycin was prescribed. An explanation for this cannot be found from our data.

For most patients, treatment duration was in line with the national guideline (7 days).^27^ This duration might have influenced treatment success as prior studies have shown increased treatment success rates with longer regimens,^28,29^ which is also incorporated in recent guidelines.^22^

### Implications for research and practice

Several clinical implications are of concern regarding the increasing antimicrobial resistance of HP. First, post-treatment HP testing may likely improve insights into resistance rates and thereby facilitate future decisions on (changes in) treatment regimens. International guidelines did incorporate confirmation of treatment success, local guidelines did not (yet).^22^ Second, the implementation of susceptibility testing for clarithromycin resistance in primary care should be considered in order to lower resistance rates.^22^ Last, the addition of bismuth to the eradication regimens could improve treatment success, especially as studies have shown that HP strains that are metronidazole and clarithromycin resistant become susceptible in case of concomitant use of bismuth.^30^ Although the international guidelines advises bismuth-containing quadruple therapy as a first-choice regimen,^22^ only non-bismuth treatments were identified in our study, which can be explained by the fact that bismuth is not available on the Dutch market.

## Patient and Public Involvement

Patients or the public were not involved in the design, or conduct, or reporting, or dissemination of our research.

## Data

The dataset relied on in this article is available from the corresponding author on reasonable request.
